# Survival-Inferred Fragility Index of Phase 3 Clinical Trials Evaluating Immune Checkpoint Inhibitors

**DOI:** 10.1001/jamanetworkopen.2020.17675

**Published:** 2020-10-23

**Authors:** David Bomze, Nethanel Asher, Omar Hasan Ali, Lukas Flatz, Daniel Azoulay, Gal Markel, Tomer Meirson

**Affiliations:** 1 Sackler Faculty of Medicine, Tel Aviv University, Tel Aviv, Israel; 2 Institute for Immunobiology, Kantonsspital St Gallen, St Gallen, Switzerland; 3 Ella Lemelbaum Institute for Immuno-Oncology, Sheba Medical Center, Ramat-Gan, Israel; 4 Department of Dermatology, University Hospital of Zurich, Zurich, Switzerland; 5 Department of Oncology, Kantonsspital St Gallen, St Gallen, Switzerland; 6 Center for Liver Diseases, Sheba Medical Center, Ramat-Gan, Israel; 7 Department of Clinical Microbiology and Immunology, Sackler Faculty of Medicine, Tel Aviv University, Tel Aviv, Israel; 8 Azrieli Faculty of Medicine, Bar-Ilan University, Safed, Israel

## Abstract

**Importance:**

In science and medical research, extreme and dichotomous conclusions may be drawn based on whether the *P* value falls above or below the threshold. The fragility index (ie, the minimum number of changes from nonevents to events resulting in loss of statistical significance) captures the vulnerability of statistics in trials with binary outcomes. There are a growing number of clinical trials of immune checkpoint inhibitors (ICIs), as well as expanding eligibility for patients to receive them. The robustness of survival outcomes in randomized clinical trials (RCTs) should be evaluated using the fragility index extended to time-to-event data.

**Objective:**

To calculate the fragility of survival data in RCTs evaluating ICIs.

**Design, Setting, and Participants:**

In this cross-sectional study, data on phase 3 prospective RCTs investigating ICIs included in PubMed from inception until January 1, 2020, were extracted. Two- or three-group studies reporting results for overall survival were eligible for the survival-inferred fragility index (SIFI) calculation, which is the minimum number of reassignments of the best survivors from the interventional group to the control group resulting in loss of significance (defined as *P* < .05 by log-rank test). For nonsignificant results, a negative SIFI was calculated by reversing the direction of reassignment (from the control group to the interventional group).

**Main Outcomes and Measures:**

Survival-inferred fragility index.

**Results:**

A total of 45 phase 3 prospective RCTs (4 of which had 3 groups, for a total of 49 groups) were identified, of which 6 (13%) investigated anti–cytotoxic T-lymphocyte–associated protein 4 (CTLA-4) agents, 25 (56%) investigated anti–programmed cell death 1 (PD-1) agents, 12 (27%) investigated anti–programmed cell death 1 ligand 1 agents, and 3 (7%) investigated the combination of anti–CTLA-4 and anti–PD-1 agents. The median SIFI was 5 (interquartile range, –4 to 12) for the intention-to-treat analysis; for these trials, the SIFI was 1% or less of the total sample size in 17 of 49 populations (35%). In 25 of the 49 intention-to-treat populations (51%), the SIFI was less than the number of censored patients in the intervention group shortly after randomization (defined as <5% of the follow-up time).

**Conclusions and Relevance:**

This study suggests that many phase 3 RCTs evaluating ICI therapies have a low SIFI for overall survival, resulting in uncertainty regarding their potential clinical benefit. Although not a definitive solution for the problems arising from dichotomization, SIFI provides an additional means of assessing and communicating the strength of statistical conclusions.

## Key Points

**Question:**

How stable are the conclusions of phase 3 randomized clinical trials of immune checkpoint inhibitors in oncology?

**Findings:**

This cross-sectional study of 45 randomized clinical trials calculated the survival-inferred fragility index and found that many oncologic trials assessing immune checkpoint inhibitors have a low survival-inferred fragility index, often less than a small fraction of the sample size and less than the number of patients censored soon after randomization.

**Meaning:**

These results challenge the robustness of many phase 3 randomized clinical trials of immune checkpoint inhibitors in oncology and address the uncertainty regarding their potential clinical benefit.

## Teaser

This cross-sectional study calculates the fragility of survival data in randomized clinical trials evaluating immune checkpoint inhibitors.

## Introduction

Immune checkpoint inhibitors (ICIs) targeting cytotoxic T-lymphocyte–associated protein 4 (CTLA-4) or programmed cell death 1 (PD-1) and programmed cell death 1 ligand 1 (PD-L1) have revolutionized cancer treatment and led to their approval as first-line therapies, either alone or in combination with chemotherapy, for many solid tumors and hematologic malignant neoplasms.^1^ However, the clinical benefit associated with ICIs cannot be generalized into a single category, as the therapeutic effectiveness varies widely across different cancer indications.^2,3,4,5,6,7^ The number of active clinical trials of ICIs is growing rapidly, along with an increased pace of accelerated approvals by the US Food and Drug Administration (FDA).^8,9^ The eligibility criteria for ICI therapy are dynamic, and results of postmarketing studies often lead to label revisions, with more changes expected to follow.^10^ Despite the popularity of ICIs and the expanding eligibility for expensive and potentially toxic treatments, the percentage of eligible patients who benefit from ICIs is decreasing.^10,11^ This gap between ICI eligibility and clinical benefit is concerning and is not fully understood.

**Figure 1.**
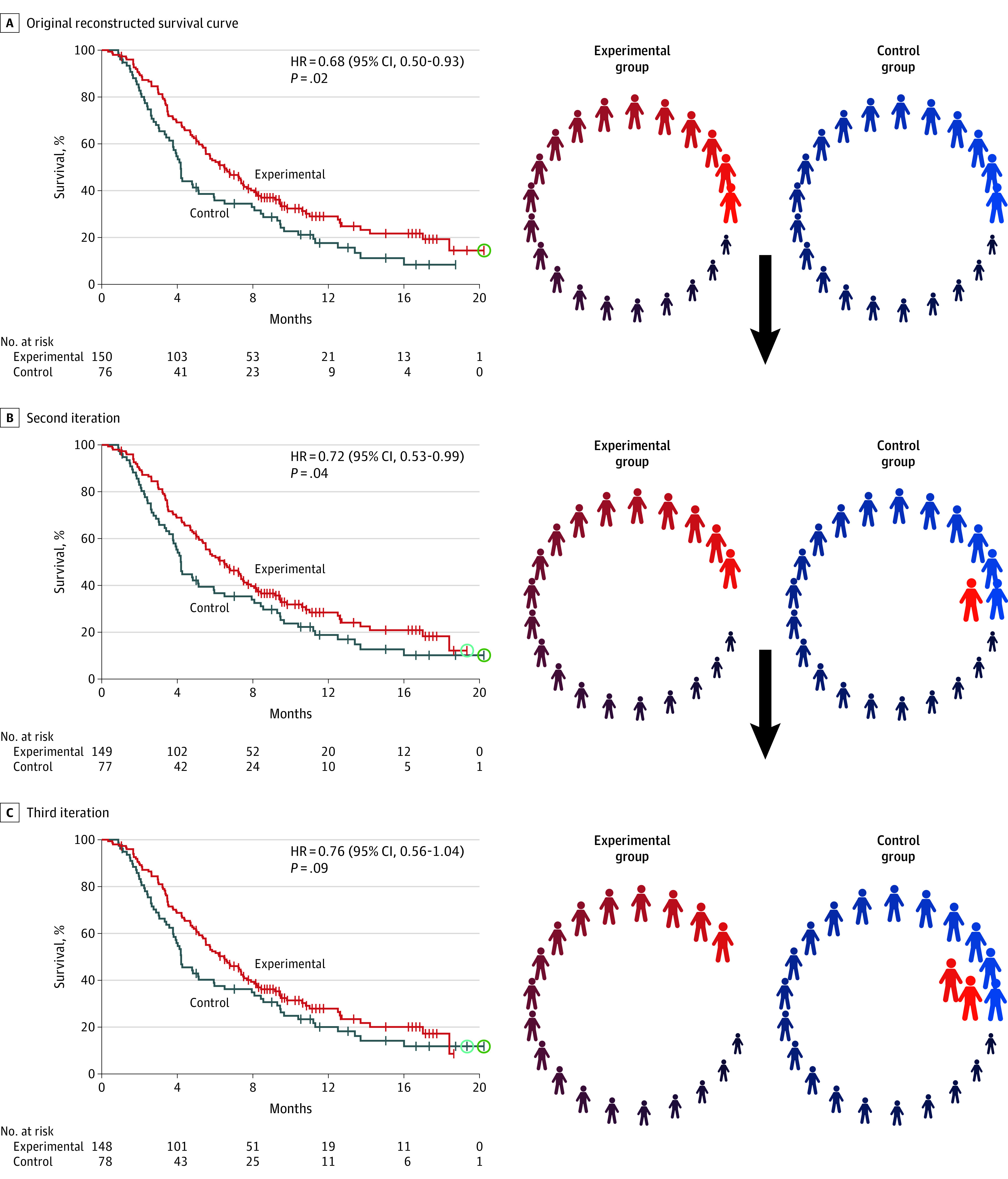
Example of Survival-Inferred Fragility Index (SIFI) Calculation of Overall Survival A, Original reconstructed survival curve. B, Second iteration of the survival curve. C, Third iteration of the survival curve. The SIFI in this example is 2, which is the iterative reassignment of the best survivors (designated by circles at the end of the survival curves) from the experimental group to the control group, until positive significance is lost (defined as α = .05 using log-rank test). HR indicates hazard ratio.

Since the introduction of the *P* value almost a century ago, reliance on a fixed cutoff serving as the gatekeeper for establishing significance in clinical trials has caused controversy.^12,13^ Statistically significant differences in outcomes using an arbitrary threshold (*P* < .05) may not be clinically relevant, especially when the estimated outcome does not offer substantial clinical benefit.^14,15^ The fragility of statistical inference can be signified by the ease with which a significant *P* value (*P* < .05) crosses over the significance threshold (*P* > .05).^16,17^ Johnson et al^18^ introduced a method to compute the fragility for survival analysis by iteratively adding artificial patients to the experimental group with events at the mean exposure time of all individuals until significance is lost. Using this method, one study has recently shown that the fragility index of time-to-event data can be used to estimate the level of confidence of positive results reported in randomized clinical trials (RCTs) leading to FDA approval of anticancer drugs.^19^ However, this approach that simulates average “virtual” patients might inflate the fragility estimate as patients at the extreme, who contribute the most to the survival curves, are disregarded. Many possible ways could be formulated to estimate the fragility of survival data. Therefore, we aimed to define a simple and intuitive fragility measure for survival analysis, based on real-life conditions, that captures the vulnerability of the data. Hence, we define the survival-inferred fragility index (SIFI) as the minimum number of reassignments of the best survivors (defined as the patients with the longest follow-up time, regardless of having an event or being censored; the worst survivors were defined as the patients with the earliest events) from the experimental group to the control group resulting in loss of significance (Figure 1). The purpose of this study is to evaluate the fragility of phase 3 RCTs comparing ICIs with control or standard treatments in a time-aware context.

## Methods

### Study Design

The cross-sectional study followed the Strengthening the Reporting of Observational Studies in Epidemiology (STROBE) reporting guideline.^20^ We searched PubMed from inception until January 1, 2020, for phase 3 RCTs of ICIs (anti–CTLA-4, anti–PD-1, and anti–PD-L1) compared with standard treatment in solid and hematologic malignant neoplasms. Key words for the literature search included *randomised*, *randomized*, *phase 3*, *phase III*, *ipilimumab*, *nivolumab*, *pembrolizumab*, *cemiplimab*, *durvalumab*, *avelumab*, and *atezolizumab*. For the fragility analysis, we included 2- or 3-group studies that reported overall survival as a primary or secondary outcome. We excluded retrospective studies, pooled studies, and post hoc subgroup analyses. When duplicate publications for the same trial were identified, we included the most updated publication. We abstracted information on trial design and the number of enrolled patients in the study. According to institutional review board policy, ethical approval is not required because no human data were included and publicly available information was used.

### Data Extraction

Overall survival data from 45 trials were extracted from Kaplan-Meier curves in the main text using DigitizeIt software (DigitizeIt) and the method by Wei and Royston^21^ using Stata, version 13.0 (StataCorp). This reverse-engineering strategy enabled us to reproduce survival time and censoring status at the individual patient level with minor differences between reconstructed and published data.^19^ We excluded publications of trials with raster images in which data extraction could not be performed directly. We separated the populations into 2 cohorts—the intention-to-treat (ITT) populations, which also included modified ITT populations, and subgroup populations.

### Statistical Analysis

The SIFI was calculated from Kaplan-Meier curves by the iterative redesignation of the best survivors from the experimental group to the control group until positive significance (defined as *P* < .05 obtained with a 2-sided log-rank test) was lost. Negative SIFI was calculated similarly, but the direction was opposite—redesignation of the best survivors from the control group to the experimental group. In addition to the default SIFI application (flipping the best survivor from the intervention group to the control group), we defined 3 alternative approaches: flipping the worst survivor from the experimental group to the control group, cloning the best survivor in the experimental group into the control group, and cloning the worst survivor in the control group to the experimental group. *P* values were calculated with the 2-sided unstratified log-rank test. The follow-up time distribution was calculated using the prodlim package in R (R Foundation for Statistical Computing). All other analyses were performed in R, version 3.5.0. The code used to calculate SIFI is available online.^22^

To provide a reference for the ranges of SIFI for various parameters of survival data, we generated synthetic survival data with the survsim package in R.^23^ The “simple.surv.sim” function was used with the Weibull distribution for both the time to event and the time to censoring. The cohort size was set to range from 100 to 1200 individuals in intervals of 100 (with a 1:1 allocation). The ancillary parameter for the events was set to 1.5, and the ancillary parameter for the censoring was set to 2, 4, 6, 8, or 10. The covariate for the effect size was set to all values between −1 and 0.2 in increments of 0.05. The β_0_ parameter for the event distribution was set to 2.0, and the β_0_ for the censoring distribution was set to 2.01.

## Results

For the period until January 1, 2020, we identified 45 phase 3 RCTs (4 of which had 3 groups, for a total of 49 groups)^2,3,4,5,6,7,24,25,26,27,28,29,30,31,32,33,34,35,36,37,38,39,40,41,42,43,44,45,46,47,48,49,50,51,52,53,54,55,56,57,58,59,60,61,62^ evaluating ICI therapies that met the inclusion criteria for survival fragility analysis. All except 2 multiple myeloma trials (4%)^2,47^ investigated solid tumors. Six trials (13%) investigated an anti–CTLA-4 agent (ipilimumab),^6,24,25,26,27,28^ 25 trials (56%) investigated anti–PD-1 agents (nivolumab and pembrolizumab),^2,3,29,30,31,32,33,34,35,36,37,38,39,40,41,42,43,44,45,46,47,48,49,50,51^ 12 trials (27%) investigated anti–PD-L1 agents (atezolizumab, avelumab, and durvalumab),^5,7,52,53,54,55,56,57,58,59,60,61^ and 3 trials (7%) investigated the combination of anti–CTLA-4 and anti–PD-1 agents (ipilimumab and nivolumab).^4,36,62^ We could not calculate the SIFI for 2 trials (CA184-002 and CA184-043)^63,64^ because of an incompatible graphical format of the Kaplan-Meier plots. The median sample size for the eligible trials was 559 (interquartile range [IQR], 418-727). The SIFI was calculated for an additional 36 subgroups (eg, PD-L1, ≥1%) in 15 trials with a median sample size of 362 (IQR, 217-486).^4,7,28,31,36,37,41,46,51,52,53,56,57,59,62^

Thirty-four of the 49 reconstructed overall survival curves in the ITT population (69%), which includes the modified ITT population, and 26 of the 36 subgroup populations (72%) were significant (*P* < .05) (Table 1).^2,3,4,5,6,7,24,25,26,27,28,29,30,31,32,33,34,35,36,37,38,39,40,41,42,43,44,45,46,47,48,49,50,51,52,53,54,55,56,57,58,59,60,61,62^ The median SIFI for ITT populations was 5 (IQR, –4 to 12) (ie, a median of 5 patients [among best survivors] reassigned to the control group was required to shift the results from significant to nonsignificant). The median SIFI for subgroup populations was 3.5 (IQR, 1-6.3) (eTable in the Supplement). In comparison, the fragility estimate for survival data by Johnson et al^18^ is unable to estimate fragility for nonsignificant results (negative fragility) and depicts higher values, with a median of 29 (IQR, 0-51) for the ITT populations and 29 (IQR, 0-43) for the subgroup populations. The absolute SIFI was less than 1% of the sample size in 17 (35%) of the 49 ITT populations and 10 (28%) of the 36 subgroup populations. Furthermore, in 25 (51%) of the 49 ITT populations and 16 (44%) of the 36 subgroup populations, the SIFI was less than the number of patients censored in the interventional group during only the first ventile (1/20th) of the follow-up time (eFigure 1 in the Supplement).

**Table 1.** SIFI of Overall Survival Calculated for 45 Phase 3 Trials Evaluating Immune Checkpoint Inhibitors in the Intention-to-Treat Populations

Intervention	Control	Tumor type	Clinical trial	Year	Sample size	HR	*P* value^a^	SIFI^b^
Anti–CTLA-4								
Ipilimumab + dacarbazine	Dacarbazine	Melanoma	CA184-024^24^	2015	502	0.72	.001	7
Ipilimumab	Placebo	Melanoma	CA184-029^25^	2016	951	0.72	.002	20
Ipilimumab	Placebo	PC	CA184-095^6^	2017	602	1.11	.47	−21
Ipilimumab + etoposide + platinum	Etoposide + platinum	SCLC	CA184-156^26^^c^	2016	954	0.94	.44	−7
Ipilimumab + paclitaxel + carboplatin	Paclitaxel + carboplatin	Squamous NSCLC	CA184-104^27^^c^	2017	749	0.91	.17	−3
Ipilimumab, 10 mg/kg	Ipilimumab, 3 mg/kg	Melanoma	CA184-169^28^	2017	727	0.84	.04	1
Anti–PD-1								
Nivolumab	Docetaxel	Squamous NSCLC	CheckMate 017^29^	2015	272	0.59	.0003	8
Nivolumab	Docetaxel	Nonsquamous NSCLC	CheckMate 057^30^	2015	582	0.73	.004	5
Nivolumab	Everolimus	RCC	CheckMate 025^31^	2019	821	0.74	.003	10
Nivolumab	Platinum	NSCLC	CheckMate 026^32^^d^	2017	423	1.02	.77	−16
Nivolumab	Placebo	GC or GEJC	ATTRACTION-2^33^	2017	493	0.63	<.0001	10
Nivolumab	Dacarbazine or carboplatin + paclitaxel	Melanoma	CheckMate 037^34^	2018	405	0.95	.59	−9
Nivolumab	Dacarbazine	Melanoma	CheckMate 066^35^	2019	418	0.46	<.0001	30
Nivolumab	Ipilimumab	Melanoma	CheckMate 067^36^	2018	631	0.65	<.0001	23
Nivolumab	Methotrexate, docetaxel, or cetuximab	HNSCC	CheckMate 141^37^	2018	361	0.68	.001	5
Nivolumab	Paclitaxel or docetaxel	ESCC	ATTRACTION-3^38^	2019	419	0.77	.015	2
Nivolumab	Docetaxel	NSCLC	CheckMate 078^39^	2019	504	0.68	.003	5
Pembrolizumab	Platinum	NSCLC	KEYNOTE-024^40^^e^	2016	305	0.60	.01	6
Pembrolizumab								
Every 2 wk	Ipilimumab	Melanoma	KEYNOTE-006^3^	2017	557	0.68	.001	15
Every 3 wk	Ipilimumab	Melanoma	KEYNOTE-006^3^	2017	555	0.68	.001	14
Pembrolizumab	Methotrexate, docetaxel, or cetuximab	HNSCC	KEYNOTE-040^41^	2018	495	0.8	.02	3
Pembrolizumab	Paclitaxel	GC or GEJC	KEYNOTE-061^42^	2018	395	0.82	.06	−1
Pembrolizumab + pemetrexed + platinum	Pemetrexed + platinum	Nonsquamous NSCLC	KEYNOTE-189^43^	2018	616	0.49	<.0001	40
Pembrolizumab + carboplatin + paclitaxel or nab-paclitaxel	Carboplatin + paclitaxel or nab-paclitaxel	Squamous NSCLC	KEYNOTE-407^44^	2018	559	0.64	.002	10
Pembrolizumab	Paclitaxel, docetaxel, or vinflunine	UC	KEYNOTE-045^45^	2019	542	0.70	.0005	9
Pembrolizumab	Cetuximab + platinum + fluorouracil	HNSCC	KEYNOTE-048^46^	2019	601	0.83	.02	2
Pembrolizumab + platinum + fluorouracil	Cetuximab + platinum + fluorouracil	HNSCC	KEYNOTE-048^46^	2019	559	0.77	.005	5
Pembrolizumab + pomalidomide + dexamethasone	Pomalidomide + dexamethasone	MM	KEYNOTE-183^47^	2019	249	1.61	.14	−47
Pembrolizumab + lenalidomide + dexamethasone	Lenalidomide + dexamethasone	MM	KEYNOTE-185^2^	2019	301	2.06	.06	−79
Pembrolizumab	Placebo	HCC	KEYNOTE-240^48^	2020	413	0.78	.04	1
Pembrolizumab + axitinib	Sunitinib	RCC	KEYNOTE-426^49^	2019	861	0.53	.0003	40
Pembrolizumab epacadostat	Pembrolizumab	Melanoma	KEYNOTE-252^50^	2019	706	1.13	.44	−43
Pembrolizumab	Platinum	NSCLC	KEYNOTE-042^51^^d^	2019	1274	0.81	.002	12
Anti–PD-L1								
Atezolizumab	Paclitaxel, docetaxel, or vinflunine	UC	IMvigor211^52^	2018	931	0.85	.02	3
Atezolizumab	Docetaxel	NSCLC	OAK^53^^f^	2018	850	0.85	.0003	12
Atezolizumab + bevacizumab + carboplatin + paclitaxel	Bevacizumab + carboplatin + paclitaxel	Nonsquamous NSCLC	IMpower150^54^^g^	2018	696	0.78	.02	4
Atezolizumab + carboplatin + etoposide	Carboplatin + etoposide	SCLC	IMpower133^55^	2018	403	0.70	.01	3
Atezolizumab + carboplatin + nab-paclitaxel	Carboplatin + nab-paclitaxel	Nonsquamous NSCLC	IMpower130^56^	2019	723	0.79	.03	2
Atezolizumab + bevacizumab	Sunitinib	RCC	IMmotion151^57^	2019	915	0.93	.71	−23
Atezolizumab + nab-paclitaxel	Nab-paclitaxel	BRCA	IMpassion130^7^	2020	846	0.86	.13	−4
Atezolizumab	Regorafenib	CRC	IMblaze370^5^	2019	180	1.19	.35	−12
Atezolizumab + cobimetinib	Regorafenib	CRC	IMblaze370^5^	2019	273	1.0	.8	−9
Avelumab	Paclitaxel or irinotecan	GC or GEJC	JAVELIN Gastric 300^58^	2018	371	1.1	.47	−13
Avelumab	Docetaxel	NSCLC	JAVELIN Lung 200^59^^d^	2018	529	0.9	.36	−6
Durvalumab	Placebo	NSCLC	PACIFIC^60^	2018	713	0.68	.001	15
Durvalumab + platinum + etoposide	Platinum + etoposide	SCLC	CASPIAN^61^	2019	537	0.73	.003	6
Anti–PD-1 + anti–CTLA-4								
Ipilimumab + nivolumab	Ipilimumab	Melanoma	CheckMate 067^36^	2018	629	0.54	<.0001	38
Ipilimumab + nivolumab	Sunitinib	RCC	CheckMate 214^4^	2019	1096	0.71	.003	18
Ipilimumab + nivolumab	Platinum doublet	NSCLC	CheckMate 227^62^	2019	1166	0.73	<.0001	24

Abbreviations: BRCA, breast cancer; CRC, colorectal cancer; CTLA-4, cytotoxic T-lymphocyte–associated protein 4; ESCC, esophageal squamous cell carcinoma; GC, gastric cancer; GEJC, gastroesophageal junction cancer; HCC, hepatocellular carcinoma; HNSCC, head and neck squamous cell carcinoma; HR, hazard ratio; MM, multiple myeloma; NSCLC, non–small cell lung carcinoma; PC, prostate cancer; PD-1, programmed cell death 1; PD-L1, programmed cell death 1 ligand 1; RCC, renal cell carcinoma; SCLC, small cell lung carcinoma; SIFI, survival-inferred fragility index; UC, urothelial carcinoma.

^a^ Calculated using 2-sided unstratified log-rank test.

^b^ Survival-inferred fragility index associated with the calculated *P* value (α = .05).

^c^ Modified intention-to-treat populations.

^d^ PD-L1 ≥ 1%.

^e^ PD-L1 ≥ 50%.

^f^ Intention-to-treat populations (n = 850).

^g^ *EGFR* or *ALK* wild-type.

**Figure 2.**
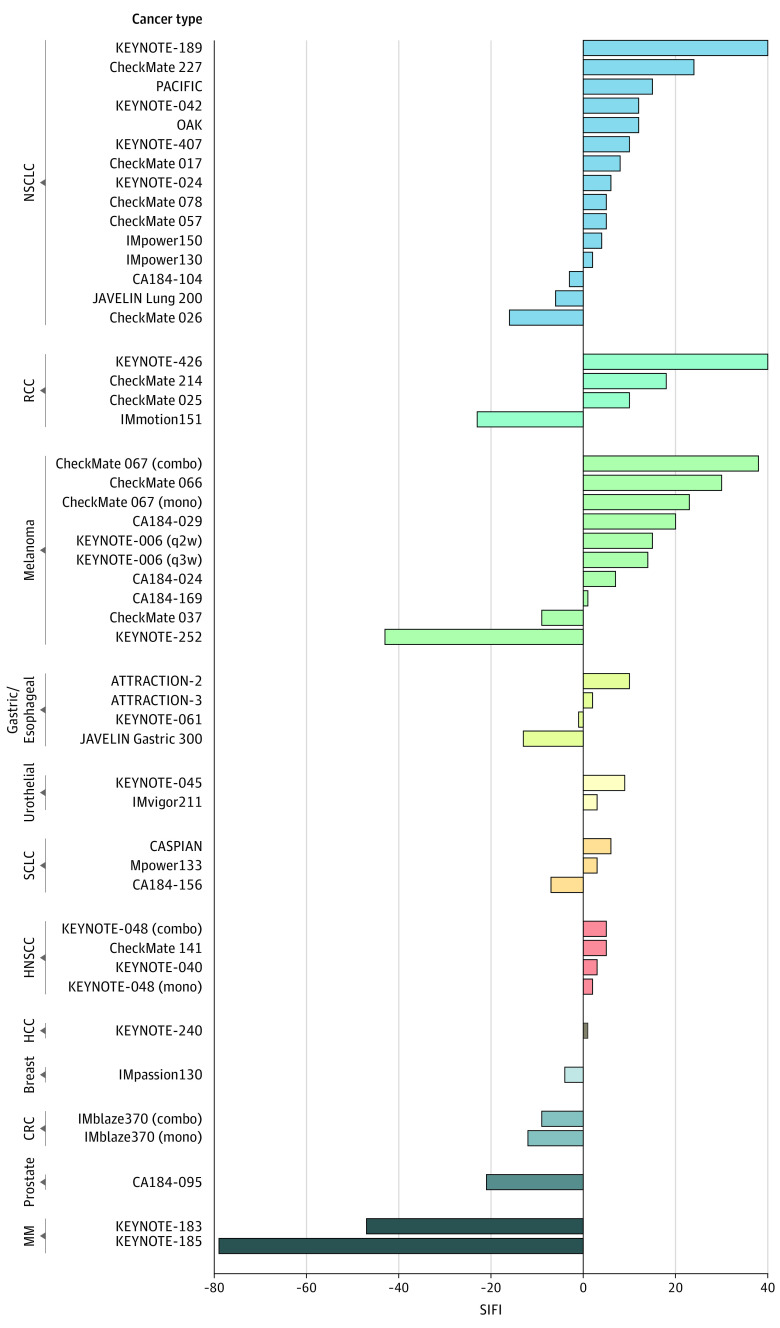
Survival-Inferred Fragility Index (SIFI) of Overall Survival in Phase 3 Randomized Clinical Trials Comparison between SIFI levels in different tumor types among the intention-to-treat populations. Trials were grouped and colored by tumor type and sorted by descending order. CRC indicates colorectal cancer; HCC, hepatocellular carcinoma; HNSCC, head and neck squamous cell carcinoma; MM, multiple myeloma; NSCLC, non–small cell lung carcinoma; RCC, renal cell carcinoma; and SCLC, small cell lung carcinoma.

**Figure 3.**
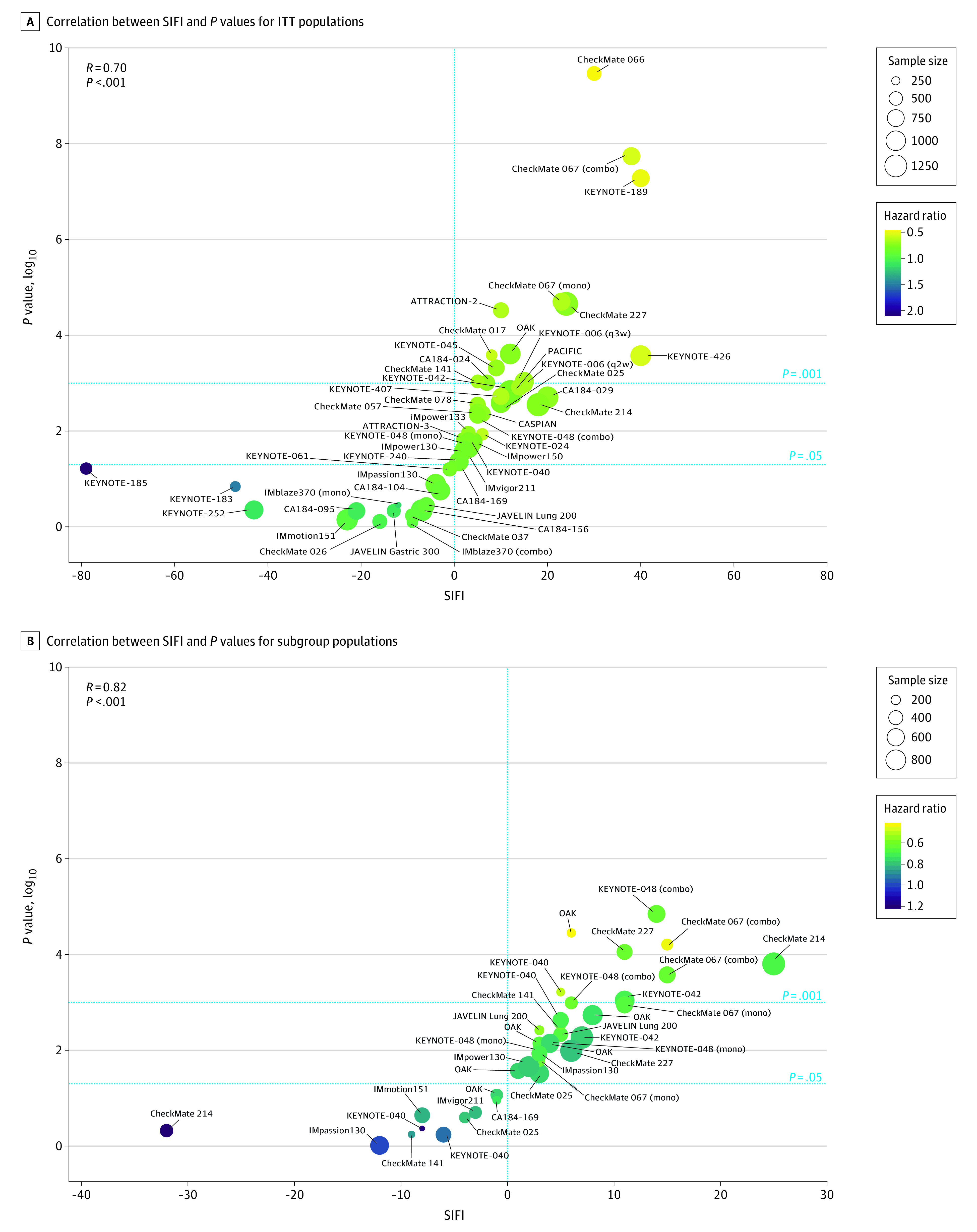
Survival-Inferred Fragility Index (SIFI) of Overall Survival in Phase 3 Randomized Clinical Trials A, Correlation between SIFI and *P* values in a logarithmic scale for the intention-to-treat (ITT) populations. B, Correlation between SIFI and *P* values in a logarithmic scale for the subgroup populations. Color bars indicate hazard ratios and circle size represents the sample size. Correlation was calculated using Pearson correlation coefficient. Horizontal lines denoting .05 and .001 *P* value thresholds are shown.

A comparison between positive SIFI levels in different tumor types among ITT populations (Figure 2) showed that non–small cell lung carcinoma, renal cell carcinoma, and melanoma had the highest values and that hepatocellular carcinoma, head and neck squamous cell carcinoma, and small cell lung carcinoma had the lowest values. Examining the association between SIFI and *P* values (in logarithmic scale) revealed a high correlation in ITT populations (*R* = 0.70; *P* < 1 × 10^−7^) and subgroup populations (*R* = 0.82; *P* < 1 × 10^−9^). However, the level of SIFI was not explained entirely by the variation in *P* values. For example, despite having relatively similar *P* values, hazard ratios, and sample sizes, the SIFI was 2-fold higher in KEYNOTE-024^40^ compared with IMpower133,^55^ and in ATTRACTION-2^33^ compared with CheckMate 067^36^ monotherapy (Table 1,^2,3,4,5,6,7,24,25,26,27,28,29,30,31,32,33,34,35,36,37,38,39,40,41,42,43,44,45,46,47,48,49,50,51,52,53,54,55,56,57,58,59,60,61,62^ Figure 3), indicating higher robustness. These examples demonstrate that statistical significance depends on the distribution of the longest-surviving patients, with more fragile studies relying on fewer patients to drive the significance, compared with less fragile studies that are associated with a higher “reserve” of patients. Similar associations between SIFI as a proportion of the population and *P* values are shown in eFigure 2 in the Supplement. To explore the potential association of longer follow-up periods with the SIFI, we identified trials that published overall survival results for earlier follow-up periods. We found that the SIFI is stable and displays only a small variation for trials at different follow-up periods (Table 2),^3,4,24,36,37,45,66,67,68,69,70^ including studies with median follow-up time more than twice as long as in the original publication. Furthermore, we explored the operating characteristics of the SIFI, including sample size, censoring rate, and effect size (eFigures 3-5 in the Supplement). Performing simulations using combinations of the parameters resulted in 15 000 synthetic time-to-event data sets. Hazard ratios ranged from 0.13 to 1.95, and the percentage of individuals censored ranged from 17.5% to 50%. The simulated results provide a reference for the ranges of the SIFI for the various parameters of survival data.

**Table 2.** Comparison of SIFI of Overall Survival Calculated for Trials in Different Follow-up Periods

Study	Tumor	Sample size	Publication year	Follow-up, median, mo	No. of events	SIFI^a^
CA184-024	Melanoma	502	2011^65^	39.0	409	5
			2015^24^	63.1	427	7
CheckMate-025	RCC	821	2015^66^	22.2	397	8
			2019^4^	45.6	567	10
CheckMate-067						
Combination therapy	Melanoma	629	2017^67^	40.2	346	37
			2018^36^	51.5	364	38
Monotherapy	Melanoma	631	2017^67^	40.2	364	23
			2018^36^	51.5	384	23
CheckMate-141	HNSCC	361	2018^68^	17.8	288	4
			2018^37^	30.6	321	5
KEYNOTE-006						
Every 2 wk	Melanoma	557	2015^69^	13.3	196	18
			2017^3^	22.5	262	15
Every 3 wk	Melanoma	555	2015^69^	13.3	203	12
			2017^3^	22.5	260	14
KEYNOTE-045	UC	542	2017^70^	13.6	333	8
			2019^45^	28.2	423	9

Abbreviations: HNSCC, head and neck squamous cell carcinoma; RCC, renal cell carcinoma; SIFI, survival-inferred fragility index; UC, urothelial carcinoma.

^a^ SIFI associated with the calculated *P* value (α = .05).

The fragility for survival data can be calculated in various ways. Overall, we calculated 4 versions of SIFI, which include reassigning patients (flip) or adding patients (clone) to the opposite group using the best survivors from the experimental group or worst survivors from the control group. A comparison of the different SIFI approaches is shown for the ITT populations in eFigure 6 in the Supplement. Compared with the default SIFI (flipping the best survivors to the opposite group) with a magnitude of 9 (IQR, 5-18) for ITT populations, the 3 alternative versions are associated with higher values in most studies. The SIFI magnitudes are 11 (IQR, 8-18) for flipping the worst survivors to the opposite group, 17.5 (IQR, 7-38.3) for cloning the best survivors to the opposite group, and 24 (IQR, 16-35) for cloning the worst survivors to the opposite group. These findings suggest that the SIFI using the version that flips the best survivors to the opposite group is the most sensitive approach for detecting the minimum changes required to overturn the conclusions.

## Discussion

In our study, we found that the statistical significance of a substantial amount of phase 3 trials of ICIs could be lost or gained with a change in assignment of very few of the best surviving patients, often less than 1% of the respective trial sample size. Although this is an arbitrary number and does not reflect a random sampling of the patients, it represents a small fraction of the population that can overturn the statistical conclusions. Also, the change in the number of patients required for fragility is often smaller than the number of patients censored in the experimental group shortly after randomization, adding further uncertainties and raising concerns about the statistical outcomes had these and other patients been assessed to their end point. Eligibility for treatment with ICIs is assessed by concluding whether results of a trial are positive or negative. Our findings demonstrate how unstable these conclusions may be, and explain, in part, the widening gap between eligibility and benefit associated with ICIs.

The original fragility index has been applied to RCTs in oncology and other areas of medicine.^17,19,71,72,73,74^ However, the original fragility index is based on binary outcomes and the Fisher exact test, which could be misleading for time-to-event data, in which the primary interest is the timing of events.^19^ Although descriptions of time-to-event fragility exist,^18,19^ to our knowledge, no previous peer-reviewed original investigations have estimated time-aware fragility index for clinical trials, including oncology trials. Also, to our knowledge, no study has evaluated negative fragility measures for survival analysis.

In general, the *P* value serves as a measure of the compatibility of collected data with a defined statistical model. In a testing framework, smaller *P* values indicate greater evidence against the null hypothesis—a conjecture of no difference between outcomes of the intervention and control groups.^75^ Undoubtedly, the *P* value plays a central role in the clinical testing of new drugs, and since the 1960s, the FDA has relied on significance testing to establish their effectiveness in the approval process.^76^ As such, nowhere is this role more important than in clinical trials, where the smallest change in the *P* value can decisively influence the drug approval process and result in trial success or failure. Consequently, passing the statistical significance threshold has become the ultimate goal, and unless an analysis is adequately prespecified, most research designs allow enough leeway to manipulate the results to claim importance.^77,78,79,80^ Therefore, reliance on *P* values falling to either side of the significance threshold can result in extreme conclusions and be misleading, especially for a low threshold such as *P* < .05. Recently, an influential commentary published in *Nature*^12^ has even called for the abandonment of the conventional threshold for statistical significance, regardless of the level (eg, *P* < .05), owing to this imposed dichotomization. However, statistical inferences are unavoidably dichotomous in many scientific fields. Most decisions in medicine are dichotomous, such as a new drug will either be approved or not, and will either be prescribed or not.^77^

This study introduces the SIFI as a novel measure that enables us to estimate the vulnerability of the statistical conclusions of clinical trials with time-to-event outcomes. This index transforms the dichotomous conclusion to a discrete variable that provides more perspective regarding the potential benefit associated with ICIs or any other intervention. The SIFI provides context to the *P* value and statistical significance, which may not necessarily be intuitive and are often poorly understood.^77^ Therefore, the SIFI translates uncertainty to a specified number that represents actual patients and events and places it on a linear scale that allows for assessment of the robustness of the results. For example, consider 2 comparable studies with similar *P* values. Although the SIFI is not a measure of effect, a trial with a high SIFI with an acceptable association with the sample size and censoring provides more robustness than a trial with a small SIFI representing a small fraction of the sample size and censoring. The latter relies on fragile evidence with higher uncertainty regarding the incompatibility with the null hypothesis. We did not define criteria for fragile vs nonfragile values, nor do we believe that a measure aimed to address the dichotomization of results by a threshold should be replaced by another. Perhaps trials involving the addition of a costly and a toxic drug to the standard treatment with a small effect size would require a higher level of robustness than trials comparing 2 drugs with similar overall properties. In contrast, concluding that statistically significant results show no real association when the fragility measure is very low is discouraged; it is equally inaccurate to claim that nonsignificant results with very small negative fragility point to an important signal. However, the SIFI allows for putting these 2 scenarios in context, expressing uncertainty and suggesting that the interpretation of their importance should be similar or, de facto, the same. In both cases, and especially for negative fragility measures, small values indicate that the true underlying effects either are negligible or lack statistical power. Nevertheless, considerations such as study design, data quality, comprehension of the underlying mechanisms, and other factors may often have more importance than statistical findings^12^ such as *P* values or fragility indices.

The default solution for improving the confidence level would be making the barrier more demanding; however, this is a suboptimal option because the chance for false-negative results increases accordingly, and it still fails to address the vulnerability of the statistics. Nevertheless, fragility corresponding to one threshold is not comparable with another, and it is reasonable to expect lower fragility measures for lower *P* value thresholds, as they are interrelated. Hence, the approach encourages using lower significance thresholds. A trial not meeting a low prespecified significance threshold (eg, *P* < .0001), with a small negative SIFI (eg, −2), may provide higher confidence in the validity of the results compared with a trial that meets a higher threshold (eg, *P* < .05) but has a low positive SIFI (eg, 2). The SIFI relative to sample size can be useful to estimate the robustness of the results, but it could be misleading for small sample sizes. Although SIFI less than 1% in many RCTs could suggest extreme fragility, small trials with less than 100 patients cannot achieve a SIFI of less than 1%, even when the results are certainly less robust. Therefore, the SIFI relative to sample size, especially for small trials, should not be interpreted alone and must be accompanied by the SIFI.

### Limitations

Several limitations of the study should be recognized. We did not address prespecified *P* value thresholds, which were allocated and controlled differently in every trial and are often much lower than .05. Instead, we used the standard α level of .05 as a common reference; therefore, some trials did not meet the prespecified threshold but resulted in a positive SIFI. Although not a strict rule by the FDA, the standard 2-trial α level is .05 but is smaller for approval based on a single trial.^76^ The analysis of overall survival was based on an unstratified log-rank test at a 2-sided significance level as a uniform statistical test for all trials; however, studies have analyzed the data differently (eg, stratified or weighted log-rank test). Therefore, small differences exist between the published *P* value and the calculated *P* value. Furthermore, we found a small discrepancy in the numbers of patients at risk published in the original publications and the reproduced curves. For 19 of the 49 populations in the trials (39%), there was no discrepancy between the published and estimated number at risk at any time point. In the time points for which discrepancy existed, we found the difference to be small, with a median of 1 patient (IQR, 1-2).

The SIFI can be calculated in various ways. Our comparison of different implementations of the SIFI demonstrates that reassigning or adding the best survivors to the opposite group provides lower fragility estimates compared with the worst survivors, for most trials. This finding indicates that the longest-surviving patients can tilt the balance between the groups more strongly compared with the shortest-surviving patients. The association of the longest survivors with the survival curves is potentially unlimited, as they are constrained only by the follow-up time, whereas the shortest-surviving patients cannot have an event before time zero. By both removing a long-time survivor from one group and adding them to the other group, the total number of patients required to pass the significance threshold is reduced compared with other techniques. This approach coincides with the essence of fragility—identifying the minimum required changes to overturn the conclusions. Furthermore, we aimed to define a simple and intuitive method that can be recreated using existing routines, is quantifiable in all conditions, and is applicable to real-world practice in which patients are randomly assigned from a pool of eligible patients. Although random variations alone can lead to large disparities in *P* values, the calculation of the SIFI is not based on random variations in the assignment of patients but on the reassignment of patients at the extreme ends of the scale. However, the random allocation of patients can lead to different proportions of the best (or worst) survivors in the groups, which may impact the outcomes. Therefore, the SIFI serves as a simple and conservative approach to reflect the fragility of the statistics. Alternatively, the mean or median survival time can be exploited in different ways to quantify the fragility^18,19^; however, this approach can underestimate the fragility if the few patients who cause most of the difference are not captured.

## Conclusions

The results of this study suggest that many phase 3 RCTs evaluating ICI therapies are fragile and challenge the confidence in rejecting or concluding superiority for these drugs compared with standard treatments. Low fragility levels express uncertainty when there is no appreciable difference between the interpretative significance of data. In contrast, high fragility levels can provide robustness and aid in binary decision-making, especially for treatments associated with high cost and toxic effects that require strong support. Interpretation of any outcome is far more complicated than just significance testing, and the SIFI as a statistical and communication tool may serve as a better starting point for discerning between science and fiction.

## Notes

Supplement. **eFigure 1.** Association Between Survival-Inferred Fragility Index and Censoring of Phase 3 Randomized Clinical Trials **eFigure 2.** Proportion of Survival-Inferred Fragility Index of Overall Survival in Phase 3 Randomized Clinical Trials **eFigure 3.** Survival-Inferred Fragility Index for Synthetic Survival Data **eFigure 4.** Relationship Between the Sample Size and Survival-Inferred Fragility Index for Synthetic Survival Data **eFigure 5.** Relationship Between the Effects Size and Survival-Inferred Fragility Index for Synthetic Survival Data **eFigure 6.** Comparison of Four Versions of the Survival-Inferred Fragility Index **eTable.** Survival-Inferred Fragility Index of Overall Survival Calculated for 15 Phase 3 Trials Evaluating Immune Checkpoint Inhibitors in Subgroup Populations **eReferences.**
