# Supplementary material for: Survival-Inferred Fragility Index of Phase 3 Clinical Trials Evaluating Immune Checkpoint Inhibitors
Source: JAMA Netw Open. 2020 Oct 23;3(10):e2017675. doi: 10.1001/jamanetworkopen.2020.17675 (PMC7584930; doi:10.1001/jamanetworkopen.2020.17675)
Supplement: Supplement. — eFigure 1. Association Between Survival-Inferred Fragility Index and Censoring of Phase 3 Randomized Clinical Trials eFigure 2. Proportion of Survival-Inferred Fragility Index of Overall Survival in Phase 3 Randomized Clinical Trials eFigure 3. Survival-Inferred Fragility Index for Synthetic Survival Data eFigure 4. Relationship Between the Sample Size and Survival-Inferred Fragility Index for Synthetic Survival Data eFigure 5. Relationship Between the Effects Size and Survival-Inferred Fragility Index for Synthetic Survival Data eFigure 6. Comparison of Four Versions of the Survival-Inferred Fragility Index eTable. Survival-Inferred Fragility Index of Overall Survival Calculated for 15 Phase 3 Trials Evaluating Immune Checkpoint Inhibitors in Subgroup Populations eReferences. [file jamanetwopen-e2017675-s001.pdf]

Supplementary Online Content

Bomze D, Asher N, Hasan Ali O, et al. Survival-inferred fragility index of phase 3 clinical trials evaluating immune checkpoint inhibitors. *JAMA Netw Open.* 2020;3(10):e2017675. doi:10.1001/jamanetworkopen.2020.17675

eFigure 1. Association Between Survival-Inferred Fragility Index and Censoring of Phase 3 Randomized Clinical Trials

eFigure 2. Proportion of Survival-Inferred Fragility Index of Overall Survival in Phase 3 Randomized Clinical Trials

eFigure 3. Survival-Inferred Fragility Index for Synthetic Survival Data

eFigure 4. Relationship Between the Sample Size and Survival-Inferred Fragility Index for Synthetic Survival Data

eFigure 5. Relationship Between the Effects Size and Survival-Inferred Fragility Index for Synthetic Survival Data

eFigure 6. Comparison of Four Versions of the Survival-Inferred Fragility Index

eTable. Survival-Inferred Fragility Index of Overall Survival Calculated for 15 Phase 3 Trials Evaluating Immune Checkpoint Inhibitors in Subgroup Populations

eReferences.

This supplementary material has been provided by the authors to give readers additional information about their work.

© 2020 Bomze D et al. *JAMA Network Open.*

Supplementary Figure 1

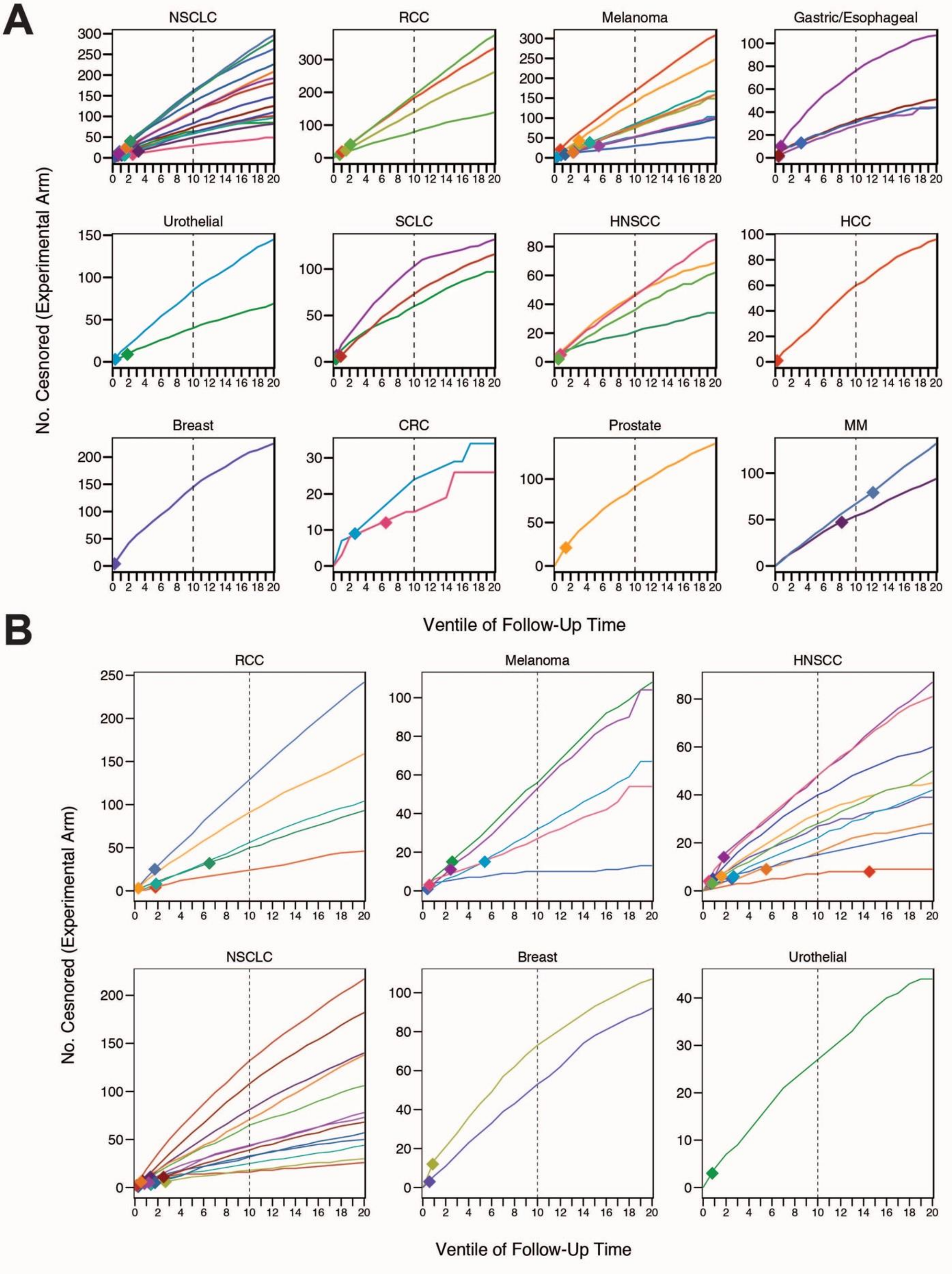

© 2020 Bomze D et al. *JAMA Network Open.*

eFigure 1. Association Between Survival-Inferred Fragility Index and Censoring of Phase 3 Randomized Clinical Trials

Number of censoring in the interventional arm for each ventile of the median follow-up time are shown for the intention-to-treat (ITT) (A) and subgroup populations (B). Trials are stratified by tumor type and colored individually. For each trial the highest ventile where the survival-inferred fragility index (SIFI) is greater than the number of patients lost to follow-up is indicated by diamond symbols. NSCLC = non-small cell lung carcinoma. RCC = renal-cell carcinoma. SCLC = small cell lung carcinoma. HNSCC = head and neck squamous cell carcinoma. HCC = hepatocellular carcinoma. BRCA = breast cancer. CRC = colorectal cancer. MM = multiple myeloma.

© 2020 Bomze D et al. *JAMA Network Open.*

Supplementary Figure 2

A

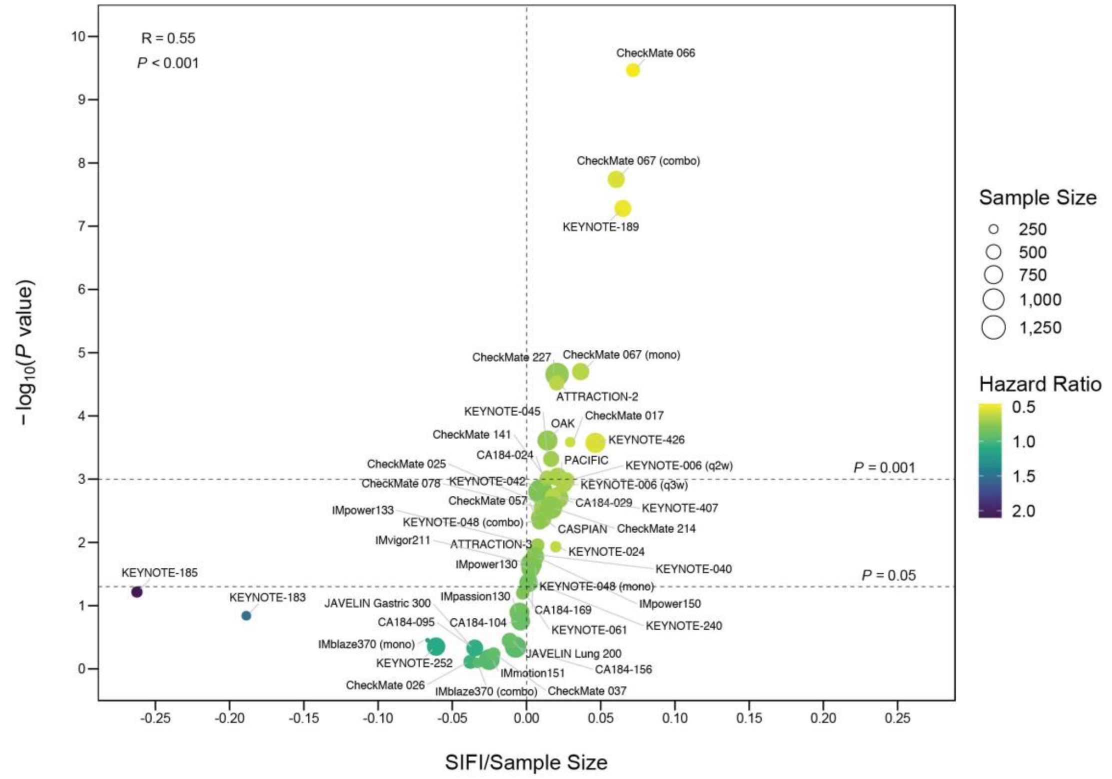

B

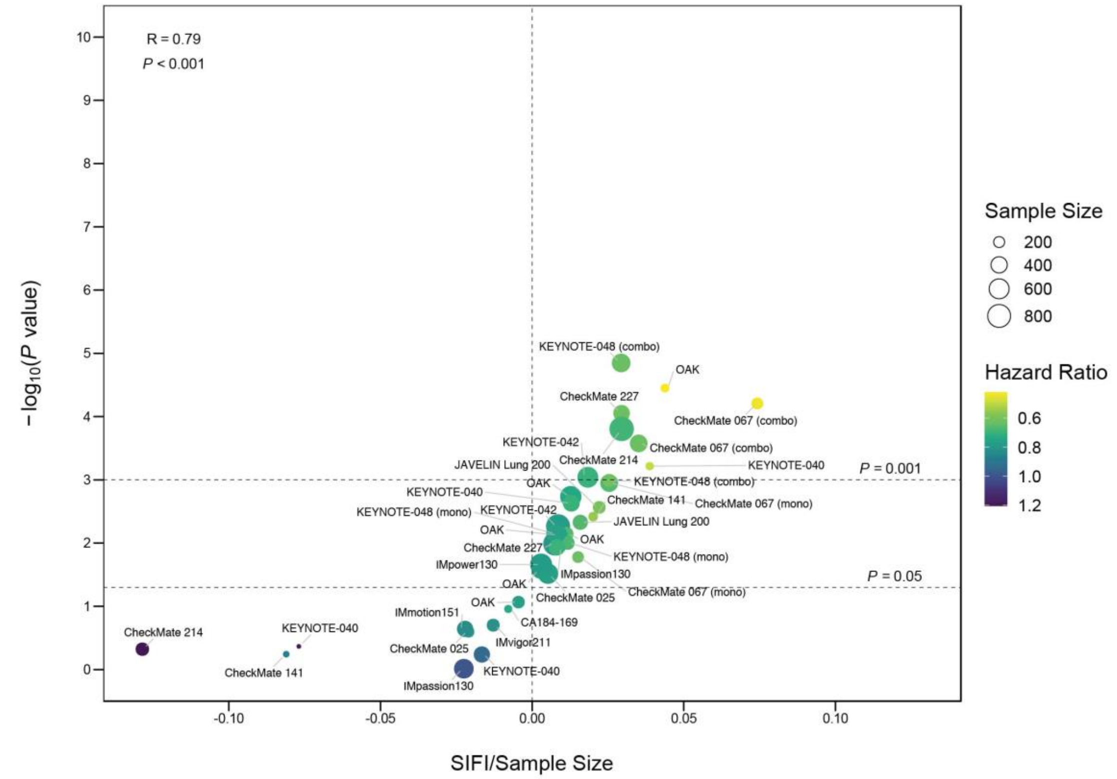

© 2020 Bomze D et al. *JAMA Network Open.*

eFigure 2. Proportion of Survival-Inferred Fragility Index of Overall Survival in Phase 3 Randomized Clinical Trials

(A) The relationship between survival-inferred fragility index (SIFI) as a proportion of the sample size and p-values in a logarithmic scale is shown for the intention-to-treat (ITT) (A) and subgroup populations (B). Color bars indicate hazard ratios (HR) and circle size represent the sample size. Correlation was calculated using Pearson's correlation coefficient. Horizontal lines denoting 0.05 and 0.001 p-value thresholds are shown.

© 2020 Bomze D et al. *JAMA Network Open.*

Supplementary Figure 3

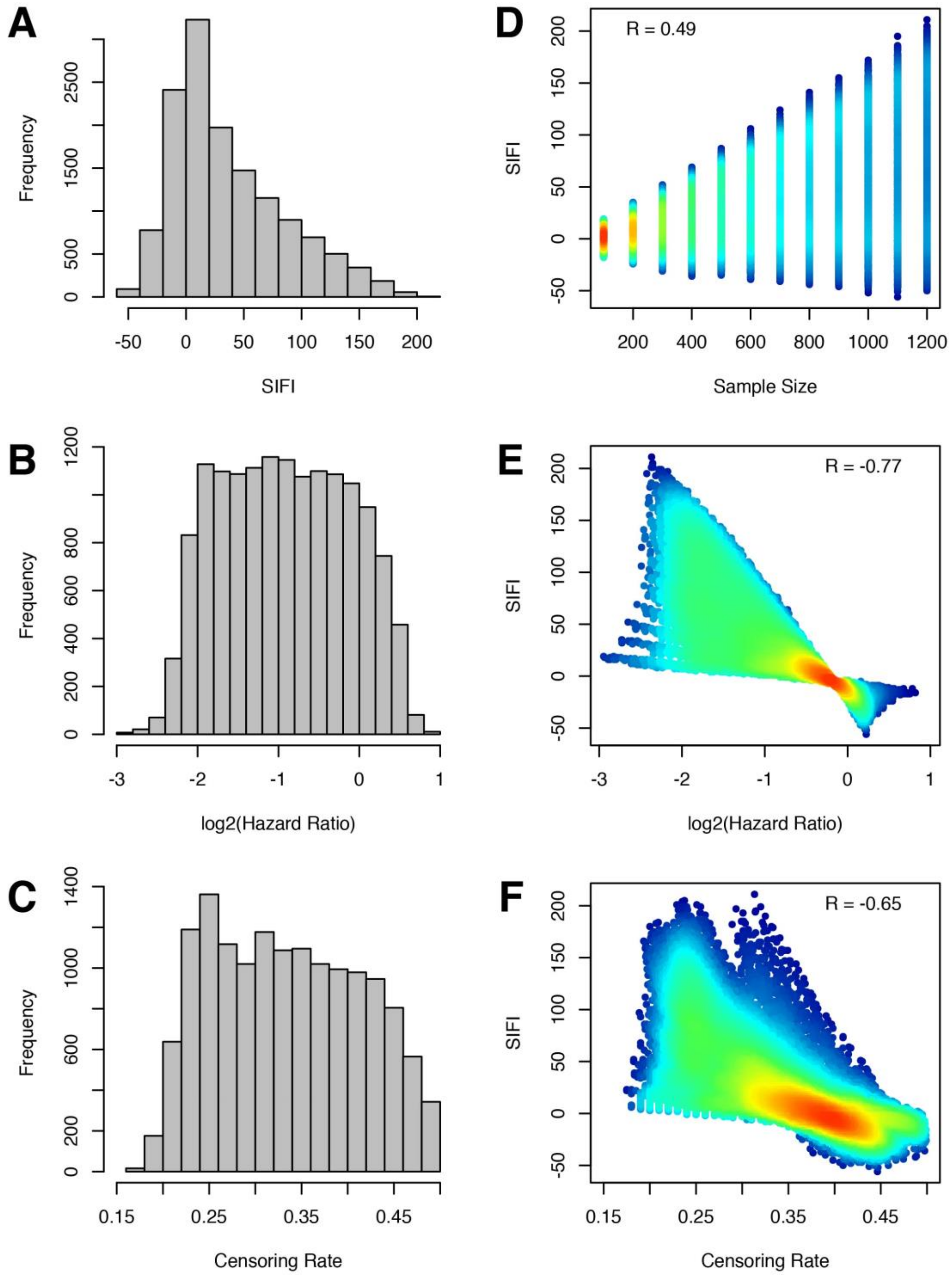

© 2020 Bomze D et al. *JAMA Network Open.*

eFigure 3. Survival-Inferred Fragility Index for Synthetic Survival Data

The distribution of survival-inferred fragility index (SIFI) (A), hazard ratios (HR) (B), and censoring rates (C) for synthetic survival data are displayed. Shown are positive correlation between SIFI and sample size (D), positive correlation between SIFI and effect size as evaluated via the HR (E), and a negative correlation between SIFI and the percent of patients censored (F). The color bars indicate the density distribution of the SIFI. Correlation was calculated using Pearson's correlation coefficient.

© 2020 Bomze D et al. *JAMA Network Open.*

Supplementary Figure 4

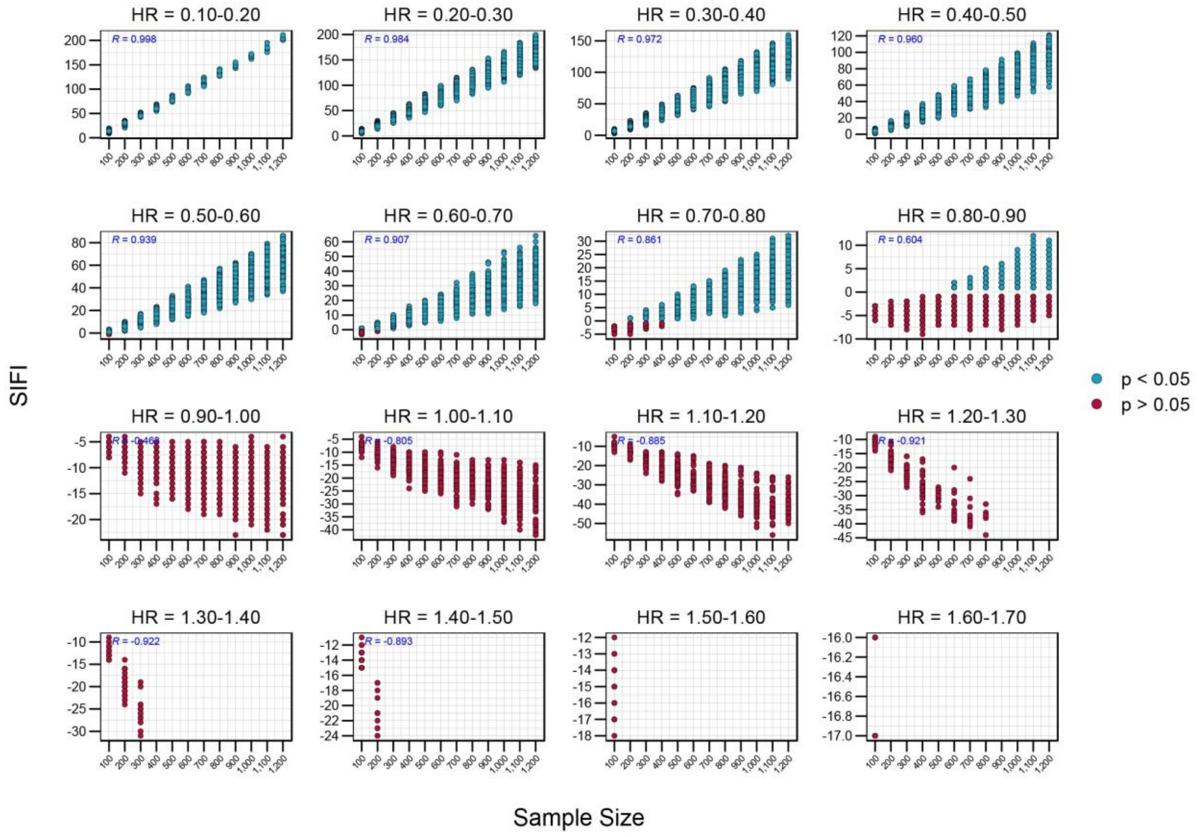

eFigure 4. Relationship Between the Sample Size and Survival-Inferred Fragility Index for Synthetic Survival Data

The survival-inferred fragility index (SIFI) for a range of sample sizes using simulated survival data are displayed. Correlation was calculated using Pearson's correlation coefficient.

© 2020 Bomze D et al. *JAMA Network Open.*

Supplementary Figure 5

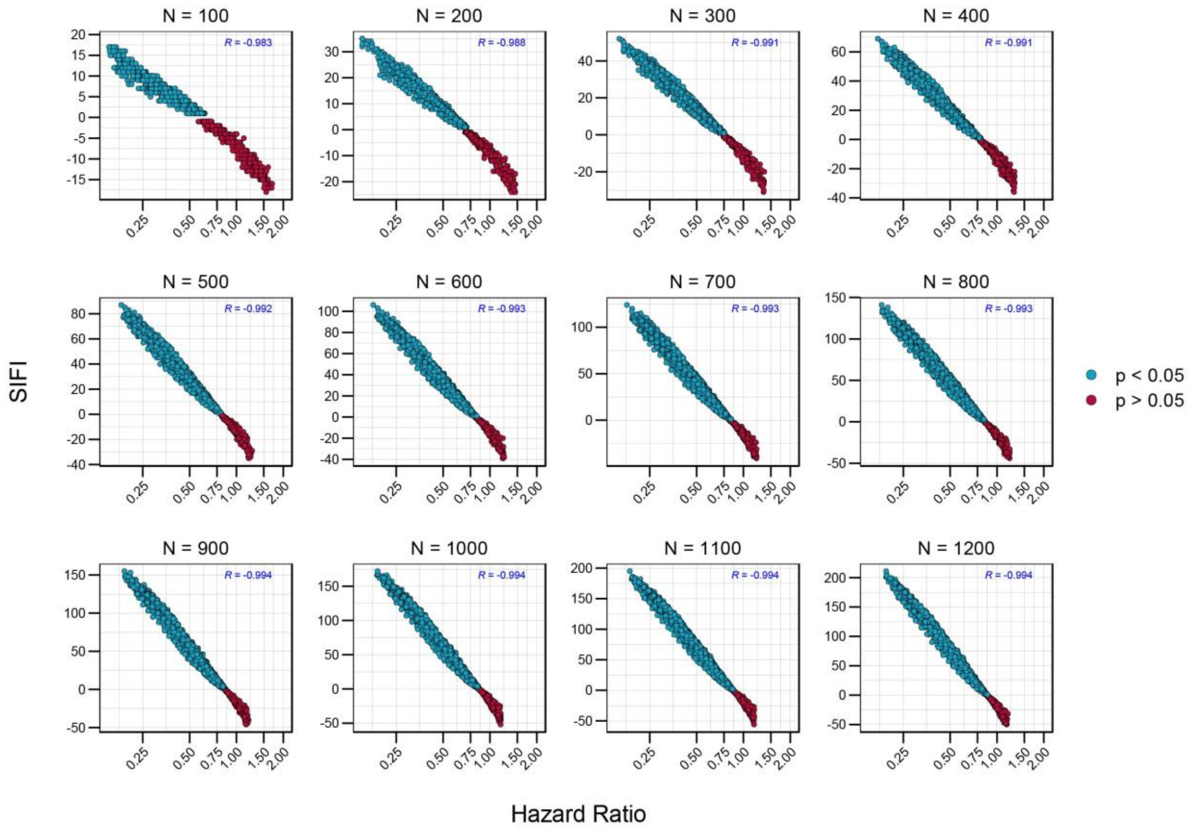

eFigure 5. Relationship Between the Effects Size and Survival-Inferred Fragility Index for Synthetic Survival Data

The survival-inferred fragility index (SIFI) for a range of hazard ratios (HR) using simulated survival data are displayed. Correlation was calculated using Pearson's correlation coefficient.

© 2020 Bomze D et al. *JAMA Network Open.*

Supplementary Figure 6

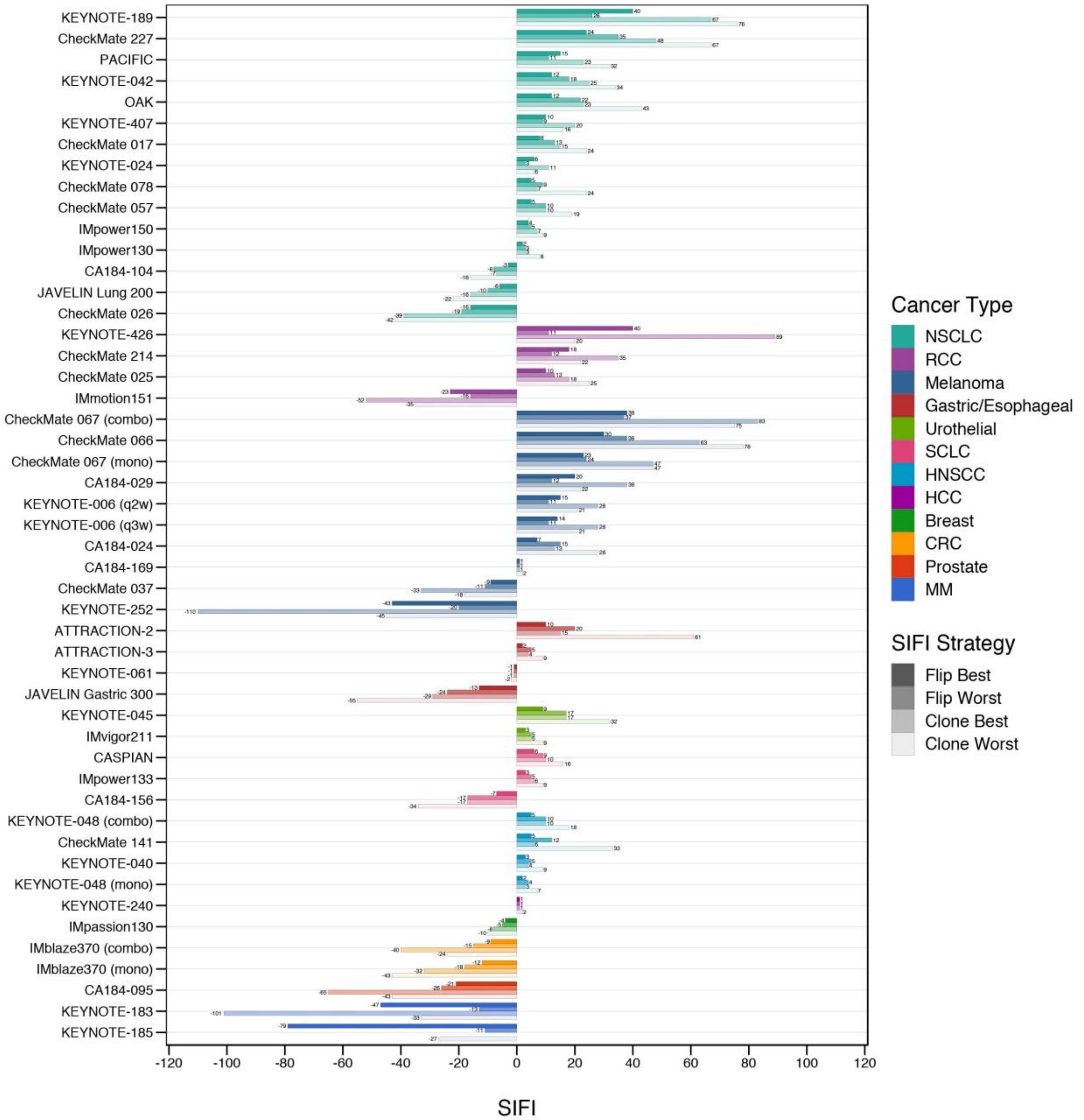

eFigure 6. Comparison of Four Versions of the Survival-Inferred Fragility Index

(A) A comparison between four versions of the survival-inferred fragility index (SIFI) for overall survival in different tumor types among the intention-to-treat (ITT) populations. Trials were grouped and colored by the tumor type and sorted by descending order. Correlation was calculated using Pearson's correlation coefficient. NSCLC = non-small cell lung carcinoma. RCC = renal cell carcinoma. SCLC = small cell lung carcinoma. HNSCC = head and neck squamous cell carcinoma. HCC = hepatocellular carcinoma. CRC = colorectal cancer. MM = multiple myeloma.

eTable. Survival-Inferred Fragility Index of Overall Survival Calculated for 15 Phase 3 Trials Evaluating Immune Checkpoint Inhibitors in Subgroup Populations

Intervention	Control	Tumor type	Clinical trial	Year	Group	Sample size	HR	Calc. p-value†	SIFI §
Anti-CTLA4									
Ipilimumab 10mg/kg	Ipilimumab 3mg/kg	Melanoma	CA184-169[1]	2017	Asympt. brain met.	127	0.71	0.11	-1
Anti-PD1									
Nivolumab	Everolimus	RCC	CheckMate 025[2]	2015	PD-L1≥1%	191	0.79	0.25	-4
Nivolumab	Everolimus	RCC	CheckMate 025[2]	2015	PD-L1<1%	575	0.77	0.03	2
Nivolumab	Ipilimumab	Melanoma	CheckMate 067[3]	2017	BRAF-	433	----	0.001	11
Nivolumab	Ipilimumab	Melanoma	CheckMate 067[3]	2017	BRAF+	198	----	0.02	3
Nivolumab	Methotrexate / docetaxel / cetuximab	HNSCC	CheckMate 141[4]	2016	PD-L1≥1%	149	0.55	0.004	3
Nivolumab	Methotrexate / docetaxel / cetuximab	HNSCC	CheckMate 141[4]	2016	PD-L1<1%	111	0.89	0.56	-9
Pembrolizumab	Platinum	NSCLC	KEYNOTE-042[5]	2019	PD-L1≥20%	818	0.77	0.005	7
Pembrolizumab	Platinum	NSCLC	KEYNOTE-042[5]	2019	PD-L1≥50%	599	0·69	0.001	11
Pembrolizumab	Cetuximab + platinum + 5FU	HNSCC	KEYNOTE-048[6]	2019	PD-L1≥1%	512	0.78	0.007	4
Pembrolizumab	Cetuximab + platinum + 5FU	HNSCC	KEYNOTE-048[6]	2019	PD-L1≥20%	255	0.61	0.01	3
Pembrolizumab + platinum + 5FU	Cetuximab + platinum + 5FU	HNSCC	KEYNOTE-048[6]	2019	PD-L1≥1%	477	0.65	0.00001	14
Pembrolizumab + platinum + 5FU	Cetuximab + platinum + 5FU	HNSCC	KEYNOTE-048[6]	2019	PD-L1≥20%	236	0.60	0.001	6
Pembrolizumab	Methotrexate / docetaxel / cetuximab	HNSCC	KEYNOTE-040[7]	2018	PD-L1≥1%	387	0.74	0.0024	5
Pembrolizumab	Methotrexate / docetaxel / cetuximab	HNSCC	KEYNOTE-040[7]	2018	PD-L1<1%	104	1.28	0.43	-8
Pembrolizumab	Methotrexate / docetaxel / cetuximab	HNSCC	KEYNOTE-040[7]	2018	PD-L1≥50%	96	0.53	0.0008	5
Pembrolizumab	Methotrexate / docetaxel / cetuximab	HNSCC	KEYNOTE-040[7]	2018	PD-L1<50%	284	0.93	0.58	-6
Anti-PD-L1									
Atezolizumab	Paclitaxel/ docetaxel/ vinflunine	UC	IMvigor211[8]	2017	IC2/3	234	0.87	0.20	-3
Atezolizumab	Docetaxel	NSCLC	OAK[9]	2018	Non-squam.	628	0.74	0.002	8
Atezolizumab	Docetaxel	NSCLC	OAK[9]	2018	Squam.	222	0.77	0.087	-1
Atezolizumab	Docetaxel	NSCLC	OAK[9]	2018	TC0 and IC0	379	0.77	0.028	1
Atezolizumab	Docetaxel	NSCLC	OAK[9]	2018	TC123 or IC123	463	0.74	0.0077	4
Atezolizumab	Docetaxel	NSCLC	OAK[9]	2018	TC23 or IC23	265	0.66	0.0075	3
Atezolizumab	Docetaxel	NSCLC	OAK[9]	2018	TC3 or IC3	137	0.40	5.6E-5	6
Avelumab	Docetaxel	NSCLC	JAVELIN Lung 200[10]	2018	PD-L1≥50%	315	0.67	0.005	5
Avelumab	Docetaxel	NSCLC	JAVELIN Lung 200[10]	2018	PD-L1≥80%	226	0.59	0.003	5
Atezolizumab + carboplatin+ nab-paclitaxel	Carboplatin + nab-paclitaxel	Non-squam. NSCLC	IMpower130[11]	2019	*EGFR* or *ALK* wild-type	679	0.79	0.02	2
Atezolizumab + nab-paclitaxel	Nab-paclitaxel	BRCA	IMpassion130[12]	2019	PD-L1≥1%	369	0.71	0.012	3
Atezolizumab + nab-paclitaxel	Nab-paclitaxel	BRCA	IMpassion130[12]	2019	PD-L1<1%	533	0.97	0.97	-12
Atezolizumab + bevacizumab	Sunitinib	RCC	IMmotion151[13]	2019	PD-L1≥1%	362	0.84	0.23	-8
Anti-PD-1 + Anti-CTLA-4									
Ipilimumab + nivolumab	Ipilimumab	Melanoma	CheckMate 067[14]	2017	BRAF+	202	------	9.8E-5	15
Ipilimumab + nivolumab	Ipilimumab	Melanoma	CheckMate 067[14]	2017	BRAF-	427	------	0.0003	15
Ipilimumab + nivolumab	Sunitinib	RCC	CheckMate 214[15]	2019	IMDC interm./ poor-risk	847	0.66	0.00017	25
Ipilimumab + nivolumab	Sunitinib	RCC	CheckMate 214[15]	2019	IMDC favor.-risk	249	1.22	0.48	-32

© 2020 Bomze D et al. *JAMA Network Open.*

	Ipilimumab + nivolumab	Platinum-doublet	NSCLC	CheckMate 227[16]	2019	PD-L1≥1%	793	0.79	0.01	6
	Ipilimumab + nivolumab	Platinum-doublet	NSCLC	CheckMate 227[16]	2019	PD-L1<1%	373	0.62	0.0001	11

NSCLC = non-small cell lung carcinoma. SCLC = small cell lung carcinoma. RCC = renal-cell carcinoma. HNSCC = head and neck squamous cell carcinoma. UC = urothelial carcinoma. BRCA = breast cancer. SIFI=Survival-inferred fragility index. † Calculated using two-sided unstratified log-rank test. § Survival inferred fragility index relates to the calculated p-value (α=0.05). IMDC=International Metastatic Renal Cell Carcinoma Database Consortium Open questions.

© 2020 Bomze D et al. *JAMA Network Open.*

eReferences

1. Ascierto PA, Del Vecchio M, Robert C, et al. Ipilimumab 10 mg/kg versus ipilimumab 3 mg/kg in patients with unresectable or metastatic melanoma: a randomised, double-blind, multicentre, phase 3 trial. *The Lancet Oncology* 2017; **18**(5): 611-22.
2. Motzer RJ, Escudier B, McDermott DF, et al. Nivolumab versus everolimus in advanced renal-cell carcinoma. *New England Journal of Medicine* 2015; **373**(19): 1803-13.
3. Hodi FS, Chiarion-Sileni V, Gonzalez R, et al. Nivolumab plus ipilimumab or nivolumab alone versus ipilimumab alone in advanced melanoma (CheckMate 067): 4-year outcomes of a multicentre, randomised, phase 3 trial. *The Lancet Oncology* 2018; **19**(11): 1480-92.
4. Ferris RL, Blumenschein Jr G, Fayette J, et al. Nivolumab vs investigator's choice in recurrent or metastatic squamous cell carcinoma of the head and neck: 2-year long-term survival update of CheckMate 141 with analyses by tumor PD-L1 expression. *Oral oncology* 2018; **81**: 45-51.
5. Mok TS, Wu Y-L, Kudaba I, et al. Pembrolizumab versus chemotherapy for previously untreated, PD-L1-expressing, locally advanced or metastatic non-small-cell lung cancer (KEYNOTE-042): a randomised, open-label, controlled, phase 3 trial. *The Lancet* 2019; **393**(10183): 1819-30.
6. Burtness B, Harrington KJ, Greil R, et al. Pembrolizumab alone or with chemotherapy versus cetuximab with chemotherapy for recurrent or metastatic squamous cell carcinoma of the head and neck (KEYNOTE-048): a randomised, open-label, phase 3 study. *The Lancet* 2019; **394**(10212): 1915-28.
7. Cohen EE, Soulières D, Le Tourneau C, et al. Pembrolizumab versus methotrexate, docetaxel, or cetuximab for recurrent or metastatic head-and-neck squamous cell carcinoma (KEYNOTE-040): a randomised, open-label, phase 3 study. *The Lancet* 2019; **393**(10167): 156-67.
8. Powles T, Durán I, Van Der Heijden MS, et al. Atezolizumab versus chemotherapy in patients with platinum-treated locally advanced or metastatic urothelial carcinoma (IMvigor211): a multicentre, open-label, phase 3 randomised controlled trial. *The Lancet* 2018; **391**(10122): 748-57.
9. Fehrenbacher L, von Pawel J, Park K, et al. Updated efficacy analysis including secondary population results for OAK: a randomized phase III study of atezolizumab versus docetaxel in patients with previously treated advanced non–small cell lung cancer. *Journal of Thoracic Oncology* 2018; **13**(8): 1156-70.
10. Barlesi F, Vansteenkiste J, Spigel D, et al. Avelumab versus docetaxel in patients with platinum-treated advanced non-small-cell lung cancer (JAVELIN Lung 200): an open-label, randomised, phase 3 study. *The Lancet Oncology* 2018; **19**(11): 1468-79.
11. West H, McCleod M, Hussein M, et al. Atezolizumab in combination with carboplatin plus nab-paclitaxel chemotherapy compared with chemotherapy alone as first-line treatment for metastatic non-squamous non-small-cell lung cancer (IMpower130): a multicentre, randomised, open-label, phase 3 trial. *The Lancet Oncology* 2019; **20**(7): 924-37.
12. Schmid P, Rugo HS, Adams S, et al. Atezolizumab plus nab-paclitaxel as first-line treatment for unresectable, locally advanced or metastatic triple-negative breast cancer (IMpassion130): updated efficacy results from a randomised, double-blind, placebo-controlled, phase 3 trial. *The Lancet Oncology* 2020; **21**(1): 44-59.
13. Rini BI, Powles T, Atkins MB, et al. Atezolizumab plus bevacizumab versus sunitinib in patients with previously untreated metastatic renal cell carcinoma (IMmotion151): a multicentre, open-label, phase 3, randomised controlled trial. *The Lancet* 2019; **393**(10189): 2404-15.
14. Wolchok JD, Chiarion-Sileni V, Gonzalez R, et al. Overall survival with combined nivolumab and ipilimumab in advanced melanoma. *New England Journal of Medicine* 2017; **377**(14): 1345-56.

© 2020 Bomze D et al. *JAMA Network Open.*

15. Motzer RJ, Rini BI, McDermott DF, et al. Nivolumab plus ipilimumab versus sunitinib in first-line treatment for advanced renal cell carcinoma: extended follow-up of efficacy and safety results from a randomised, controlled, phase 3 trial. *The Lancet Oncology* 2019; **20**(10): 1370-85.
16. Hellmann MD, Paz-Ares L, Bernabe Caro R, et al. Nivolumab plus ipilimumab in advanced non–small-cell lung cancer. *New England Journal of Medicine* 2019; **381**(21): 2020-31.

© 2020 Bomze D et al. *JAMA Network Open.*
